# Membrane Thinning Induces Sorting of Lipids and the Amphipathic Lipid Packing Sensor (ALPS) Protein Motif

**DOI:** 10.3389/fphys.2020.00250

**Published:** 2020-04-16

**Authors:** Niek van Hilten, Kai Steffen Stroh, Herre Jelger Risselada

**Affiliations:** ^1^Leiden Institute of Chemistry, Leiden University, Leiden, Netherlands; ^2^Institute for Theoretical Physics, Georg August University Göttingen, Göttingen, Germany

**Keywords:** membrane thinning, lipid sorting, protein sorting, membrane deformation, membrane curvature, lipid packing, curvature sensing

## Abstract

Heterogeneities (e.g., membrane proteins and lipid domains) and deformations (e.g., highly curved membrane regions) in biological lipid membranes cause lipid packing defects that may trigger functional sorting of lipids and membrane-associated proteins. To study these phenomena in a controlled and efficient way within molecular simulations, we developed an external field protocol that artificially enhances packing defects in lipid membranes by enforcing local thinning of a flat membrane region. For varying lipid compositions, we observed strong thinning-induced depletion or enrichment, depending on the lipid's intrinsic shape and its effect on a membrane's elastic modulus. In particular, polyunsaturated and lysolipids are strongly attracted to regions high in packing defects, whereas phosphatidylethanolamine (PE) lipids and cholesterol are strongly repelled from it. Our results indicate that externally imposed changes in membrane thickness, area, and curvature are underpinned by shared membrane elastic principles. The observed sorting toward the thinner membrane region is in line with the sorting expected for a positively curved membrane region. Furthermore, we have demonstrated that the amphipathic lipid packing sensor (ALPS) protein motif, a known curvature and packing defect sensor, is effectively attracted to thinner membrane regions. By extracting the force that drives amphipathic molecules toward the thinner region, our thinning protocol can directly quantify and score the lipid packing sensing of different amphipathic molecules. In this way, our protocol paves the way toward high-throughput exploration of potential defect- and curvature-sensing motifs, making it a valuable addition to the molecular simulation toolbox.

## 1. Introduction

Biological membranes are highly dynamic and heterogeneous lipid bilayer barriers that physically separate the functional compartments of cells (McMahon and Gallop, 2005). The role of membrane curvature in the spatial organization of membrane-associated proteins (Hatzakis et al., 2009; Bhatia et al., 2010; Antonny, 2011; Singh et al., 2012; Nguyen et al., 2017; Nepal et al., 2018; Bhaskara et al., 2019) and the membrane's local lipid composition (Derganc, 2007; Jiang and Powers, 2008; Sorre et al., 2009; Tian and Baumgart, 2009; Baumgart et al., 2011; Callan-Jones et al., 2011; Baoukina et al., 2018; Harayama and Riezman, 2018; Woodward et al., 2018) (and *vice versa*!) are well-established. One important driving force that underlies these phenomena are lipid packing defects. Positive membrane curvature (the outer leaflets of vesicles) and surface tension both increase the exposure of alkyl chains to solvents via increased formation of packing defects. This results in an increased attractive stress at the oil-water interface, as evident from lateral pressure profiles (see Figure S1A and Ollila et al., 2009; Nepal et al., 2018). Proteins or lipids can reduce or even nullify the energetic cost associated with defect formation depending on their intrinsic molecular shape (Risselada and Marrink, 2009; Pinot et al., 2014; Baoukina et al., 2018) and amphipathicity (Ouberai et al., 2013; Vanni et al., 2013). They consequently reduce the elastic energy associated with membrane bending. Therefore, provided that the concomitant enthalpic gain is bigger than the entropic loss of demixing, lipids or proteins are effectively attracted toward the curved region of the membrane (i.e., curvature sensing).

Although the field's focus has been mostly on membrane curvature, lipid packing defects are a more general phenomenon. Due to the heterogeneity of biological membranes, there are many irregularities that may cause disturbances of the local lipid packing in a similar fashion to curvature; e.g., the edges of lipid nanodomains (“rafts”) (Schäfer and Marrink, 2010; Nickels et al., 2015; Belička et al., 2017; Park and Im, 2018), membrane junctions [e.g., membrane fusion intermediates (Risselada et al., 2014; Tsai et al., 2014; Smirnova et al., 2019), and transmembrane proteins (Lee, 2003; Corradi et al., 2018)]. In these three examples, the difference in chemical potential imposed by the membrane inhomogeneity alters its surrounding membrane environment, and this has interesting consequences for biological functionality.

To date, simulation tools that can study and quantify lipid packing defect-induced sorting in both a controlled and computationally efficient way are still lacking. Existing setups/protocols are based on perforated vesicles (Risselada and Marrink, 2009), membrane tethers (Baoukina et al., 2018), or buckled membranes (Elías-Wolff et al., 2018, 2019). However, all of these setups are based on local differences in membrane curvature, and they involve the simulation of relatively large, complex, and computationally expensive systems. Local differences in lipid packing can alternatively result from local differences in membrane thickness rather than membrane curvature. Conveniently, a strong but gradual change in membrane thickness can be confined to a few nanometers only. This results in a very steep “packing defect” gradient. Therefore, a sensing protocol based on local differences in thickness has the potential to (i) substantially reduce the size of the simulation box and (ii) better emphasize differences in defect sensing because of a larger sorting force (steep gradient).

To this aim, we have designed a protocol that exerts an external force on a user-defined section of a flat bilayer membrane to locally decrease its thickness. When a membrane is squeezed, lipid tails get exposed to the water phase because they must expand laterally (xy) to conserve their volume with a decreasing z-component. These lipid packing defects are comparable to the scenario imposed by (i) surface tension, (ii) protein-induced negative hydrophobic mismatch (Milovanovic et al., 2015), (iii) and the outer monolayers of vesicles (positive membrane curvature). This allows one to study the properties of a membrane that, in terms of lipid packing defects, *behaves like* it is positively curved but that has an actual membrane curvature of zero. We have illustrated that our thinning protocol can be used as a convenient proxy for scoring positive curvature-induced sorting and sensing in the absence of its conjugated negative membrane curvature and in a much smaller simulation box. Moreover, our thinning protocol is extremely adaptive, tunable, and easy to set up. As a proof of principle, we have demonstrated strong thinness-induced sorting of four biorelevant lipids and the amphipathic ALPS protein motif; this is in line with theory as well as previous simulations and experiments.

## 2. Methods

### 2.1. Simulation Details

All simulations were performed using the MARTINI force field for coarse-grained (CG) molecular dynamics (MD) (Marrink et al., 2007), and the version used was version 2.0 in GROMACS 2019.3 (Abraham et al., 2015). A 20 fs time step was used. The compressibility was set to 4.5 · 10^-5^ bar^−1^. The temperature was coupled to 310 K by the velocity rescaling thermostat (Bussi et al., 2007) (τ_T = 1 ps). Van der Waals and coulomb interactions were described by shifted potentials that gradually switched off with interaction distances exceeding 0.9 and zero, respectively. For both potentials, a 1.2 nm neighbor list cut-off was used. The neighbor list was updated every 10 simulation steps.

### 2.2. Thinning Protocol

**Figure 1:**
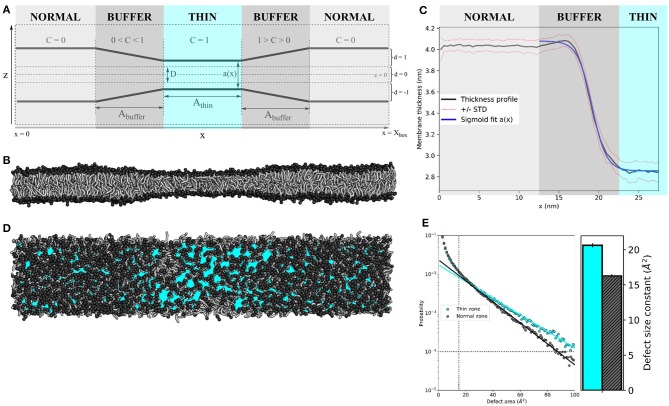
Membrane thinning induces lipid packing defects. **(A)** Schematic representation of the thinning protocol setup. Parameters correspond to those in Equations (1–4). **(B)** Side view of applying the thinning protocol to a 55 × 10 nm^2^ POPC membrane. **(C)** The average thickness profile for the same 55x10 nm^2^ POPC membrane (black line), symmetrized around the center of the thin zone. Red lines indicate the one standard deviation (STD) interval. Blue line is the sigmoid fit *a*(*x*) (see Equation 4). **(D)** Top view of the 55 × 10 nm^2^ POPC membrane. Cyan patches depict lipid packing defects (visualized by PackMem (Gautier et al., 2018). **(E)** Quantification of lipid packing defects in the thin (cyan) and normal zones (black). The block-averaged defect size constants in the bar plot (right) equate to the inverse of the slope of the linear fit to the probability distribution (left). The vertical and horizontal dotted lines indicate the minimal values for curve fitting (area *A* = 15 Å^2^ and probability *p*(*A*) = 10^-4^, as recommended by the PackMem authors (Gautier et al., 2018).

A module was added to the GROMACS 2019.3 source code to exert an inward-directed harmonic force *F*_z_ (with user-defined force constant *k*, see Equation 1) on lipid tail beads within a lateral section of a lipid bilayer, resulting in effective thinning of the membrane in that area. See Figure 1A for a schematic representation of the protocol.

(1) $$\begin{array}{l} {\vec{F}}_{\text{z}}=d\cdot C\cdot k\cdot \left(\frac{D}{2}-|z|\right) \end{array}$$

The value *D* determines the minimal membrane thickness: *F*_z_ only acts on lipid tail beads that are more than $\frac{D}{2}$ from the center (which is at *z* = 0) of the bilayer, as controlled by *d*. Constant *d* also directs the force to the center of the bilayer.

(2) $$\begin{array}{l} d=\left\{ \begin{array}{cc} 1 & \text{if }z>\frac{D}{2}\;(\text{upper leaflet})\\ 0 & \text{if }-\frac{D}{2}<z<\frac{D}{2}\;(\text{within minimal membrane}\\ \; & \;\text{thickness})\\ -1 & \text{if }z<-\frac{D}{2}\;(\text{lower leaflet}) \end{array}\right. \end{array}$$

The scaling factor *C* depends on the particle's x-coordinate. The simulation box is split into three zones–the centered “thin” zone, with user-defined length A_thin, flanked by two “buffer” zones with user-defined length A_buffer and the “normal” zones–that are connected through the periodic boundaries in the x-direction (Figure 1A). Constant *C* linearly scales from 1 (in the thin zone) to 0 (in the normal zone).

(3) $$\begin{array}{l} C=\left\{ \begin{array}{cc} 1 & \text{if }\frac{X_{\text{box}}-A_{\text{thin}}}{2}\leq x\leq \frac{X_{\text{box}}+A_{\text{thin}}}{2}\;(\text{thin zone})\\ \frac{\left|\frac{X_{\text{box}}+A_{\text{thin}}}{2}+A_{\text{buffer}}-x\right|}{A_{\text{buffer}}} & \text{if }\frac{X_{\text{box}}+A_{\text{thin}}}{2}<x\leq \frac{X_{\text{box}}+A_{\text{thin}}}{2}+A_{\text{buffer}}\;(\text{buffer zone})\\ \frac{\left|\frac{X_{\text{box}}-A_{\text{thin}}}{2}-A_{\text{buffer}}-x\right|}{A_{\text{buffer}}} & \text{if }\frac{X_{\text{box}}-A_{\text{thin}}}{2}-A_{\text{buffer}}<x\leq \frac{X_{\text{box}}-A_{\text{thin}}}{2}\;(\text{buffer zone})\\ 0 & \text{if }x<\frac{X_{\text{box}}-A_{\text{thin}}}{2}-A_{\text{buffer}}\;(\text{normal zone})\\ 0 & \text{if }x>\frac{X_{\text{box}}+A_{\text{thin}}}{2}+A_{\text{buffer}}\;(\text{normal zone}) \end{array}\right. \end{array}$$

For all simulations in this paper, the thinning parameters were set to *k* = 20 kJ nm^−2^ mol^−1^, *D* = 1 nm and *A*_thin_ = *A*_buffer_ = 10 nm. We exaggerated the size of the normal and thin zones for the sake of visual clarity.

### 2.3. Lipid Mixing Simulations

The *insane* python script (Wassenaar et al., 2015) was used to generate a 50 × 10 × 10 nm^3^ simulation box with bilayers of varying composition in the XY-plane: pure 16:0–18:1 PC (POPC), 70 mol% POPC with 30 mol% 16:0–18:1 PE (POPE), 70 mol% POPC with 30 mol% cholesterol, 70 mol% POPC with 30 mol% lysophosphatidylcholine (LysoPC, PPC in MARTINI) or 70 mol% POPC with 30 mol% 16:0–18:2 PC (PLiPC, PIPC in MARTINI) in explicit MARTINI water. After steepest descent minimization, a 2 μs NPT equilibration was performed with semiisotropic pressure coupling by the Berendsen barostat (Berendsen et al., 1984) to a reference pressure of 1 bar. Coupling of the membrane to overall tensionless conditions via a single pressure bath is ambiguous because of the heterogeneous nature of the system. It is important that the simulation box can accommodate for the extra membrane area that is created when the thin zone is being compressed. Our goal was to achieve tensionless conditions for the normal zone. To this aim, for each lipid composition, short runs with Berendsen surface tension coupling (τ_P = 2 ps) to lateral pressures ranging from 25 to 50 mN/m were performed to find the surface tension at which the membrane thickness of the normal zones matches the thickness of the corresponding tensionless membranes obtained from a normal unbiased simulation (Figure S2). At the respective calibrated surface tension coupling settings, the membranes slightly expand in the x-direction to an equilibrium box length of 55 nm for pure POPC (see Table S1 for details). A reference pressure of 1 bar was used in the z-direction. We performed five replicas (with random initial velocities) of 20 ns equilibration followed by 4 μs production runs for each lipid composition.

### 2.4. Umbrella Sampling of the ALPS Protein Motif

A CG model of the amphipathic lipid packing sensor (ALPS) motif of the ArfGAP1 protein was built from the atomistic MD-refined structure by González-Rubio et al. (2011) with the *martinize* python script (Monticelli et al., 2008). Based on the peptide's hydrogen bonding pattern, a helical secondary structure was assigned to all residues by DSSP (Kabsch and Sander, 1983). The backbone angle parameters of the central region of the peptide (GWSSFTTG) were relaxed to MARTINI's default values for loop regions (*k* = 20 kJ rad^−2^ mol^−1^, angle = 96°) to allow for some flexibility, which is known to play a role in membrane interactions (González-Rubio et al., 2011). Still, we should note that the MARTINI model does not fully capture changes in the secondary structure. Since our focus was on ALPS' amphipathicity, we deemed an aphipathic helical structure to sufficiently capture its characteristics. However, our general method is not limited to coarse-grained simulations only, and, in principle, allows for the incorporation of dynamic changes in the secondary structure via atomistic simulations.

After energy minimization, the CG ALPS peptide was placed in the middle of the thin zone of the aforementioned 55 × 10 nm^2^ pure POPC membrane. Following 200 ns of NPT equilibration (protein restrained), the center of mass (COM) of the peptide was pulled in the x-direction from the center of the thin zone (*x* ≈ 27.5 nm) to the center of the normal zone (*x* ≈ 5.5 nm), while leaving the y- and z-coordinates unrestrained. From this trajectory, 111 frames were extracted with the x-coordinate decreasing with 0.2 nm steps. Each of these frames was NPT equilibrated for 50 ns and run for 100 ns with a harmonic potential (*k* = 10^3^ kJ nm^−2^ mol^−1^) to restrict the x-coordinate of the peptide's COM to that of the respective umbrella window. The free energy profile *F*(*x*) was calculated from the 111 umbrella sampling production run data using the weighted histogram analysis method (WHAM) (Kumar et al., 1992) to unbias the probability distributions, as implemented in GROMACS (Hub et al., 2010).

## 3. Results

### 3.1. Membrane Thinning Causes Lipid Packing Defects

The setup of the thinning protocol is depicted schematically in Figure 1A (see Method section for details). Applying this protocol to a 55 × 10 nm^2^ pure POPC bilayer (*k* = 20 kJ nm^−2^ mol^−1^, *D* = 1 nm and *A*_thin_ = *A*_buffer_ = 10 nm) yields an hourglass-shaped membrane (Figure 1B). We measured the membrane thickness (the distance between amine headgroup beads of opposing membrane leaflets) over 10 μs of simulation and symmetrized the data around the center of the simulation box (Figure 1C). The average thickness was 4.03 ± 0.06 nm for the normal zone, which closely matches the thickness of a well-equilibrated MARTINI POPC membrane without thinning (4.08 ± 0.05 nm, see Table S1). The average thickness of the thin zone was 2.85 ± 0.09 nm. At the edge of the buffer zone (around *x* = 15 nm), we observed a slight overshoot (1Å) in the thickness profile. Such overshoots were observed before in the context of protein nanopores with a mismatched hydrophobic thickness (Marelli, 2012; Garcia-Fandiño et al., 2016). The thickness profile in the buffer zone as a function of the x-coordinate can be described by a sigmoid function *a*(*x*) (see Equation 4), which neatly falls within one standard deviation (STD) interval (Figure 1C). For our setup, *L* = −1.22 nm, κ = −19.14 nm^−1^, *x*_0_ = 1.11 nm, and *b* = 4.08 nm.

(4) $$\begin{array}{l} a\left(x\right)=\frac{L}{1+e^{-\kappa \left(x-x_{0}\right)}}+b \end{array}$$

We used PackMem (Gautier et al., 2018) to visualize and quantify the lipid packing defects that arise upon membrane thinning. In Figure 1D, cyan patches depict regions of the membrane where the hydrophobic lipid tails are exposed to water. Quantification was performed on 4,000 snapshots from a 400 ns simulation of a 10 × 10 nm^2^ POPC membrane with and without applying the thinning protocol (PackMem's recommended settings). As expected, the block-averaged defect size constant is higher in the thin membrane than in the normal membrane (Figure 1E; 20.62 ± 0.31 vs. 16.28 ± 0.23 Å^2^, respectively). This indicates that there is a significantly higher probability of finding greater lipid packing defects in a thin POPC membrane than in normally thick POPC membrane.

### 3.2. Thinness-Induced Lipid Sorting

**Figure 2:**
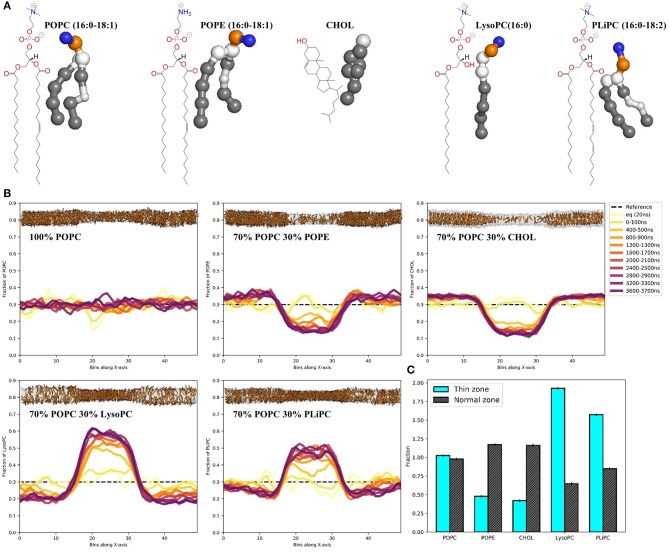
Membrane thinning induces lipid sorting. **(A)** Chemical structures and MARTINI's coarse-grained representations of POPC, POPE, CHOL, LysoPC, and PLiPC. **(B)** Histograms of the POPC, POPE, CHOL, LysoPC, and PLiPC content in 50 bins along the x-axis over 4 μs of simulation. Data are averages over five replica runs. As a reference, the black dashed line depicts the initial distribution of 30 mol%. Membrane snapshots show the final configuration, with 70 mol% POPC in transparent gray and 30 mol% of mixed-in lipid in orange. The full trajectories are provided as movies in the Supplementary Material. **(C)** Quantification of lipid mixing after 4 μs of simulation for every lipid composition in the thin zone (cyan) and the normal zone (black). Data is normalized to 30 mol%.

With the thinning protocol in hand, we proceeded to test whether the induced lipid packing defects in a POPC membrane would lead to sorting when mixing in other lipids (Figure 2A). Starting with random distributions of 30 mol% POPE, cholesterol, LysoPC or PLiPC, we tracked the lipid positions over five replicas of 4 μs simulations (Figure 2B, see trajectory movies in Supplementary Material). We found that both POPE and cholesterol partition away from the thin zone of the membrane: both concentrations more than halved (Figure 2C). In contrast, we found that both LysoPC (only one lipid tail) and PLiPC (doubly unsaturated) were strongly enriched in the thin zone of our squeezed membrane (Figure 2C).

### 3.3. Thinness-Induced Sorting of the Curvature Sensing ALPS Protein Motif

Finally, we investigated protein sorting along the thinning gradient of our POPC membrane. One of the best studied curvature sensing protein motifs is the α-helical ALPS (Bigay et al., 2005; Drin et al., 2007; Mesmin et al., 2007; González-Rubio et al., 2011; Vanni et al., 2014; Doucet et al., 2015). This 20–40 residue motif comprises regularly distributed hydrophobic residues that can favorably complement the lipid packing defects that arise at positive membrane curvature (Vanni et al., 2013). Recent simulations of a similar ALPS motif showed effective sensing of defects up to ≈ 115 Å^2^ (Wildermuth et al., 2019), which is exactly the regime of defect sizes that our thinning protocol can controllably induce. Hence, we set out to quantify the attractive force acting on the ALPS peptide toward the lipid packing defects in the thin membrane region.

**Figure 3:**
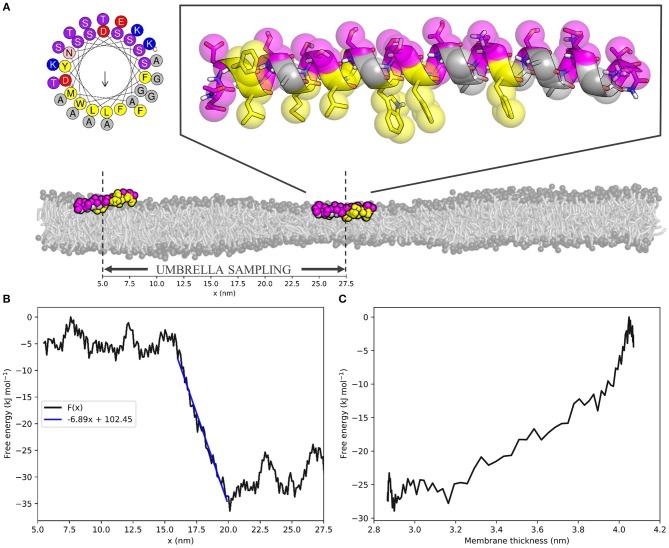
The ALPS peptide is attracted toward the thin zone of the membrane. **(A)** Top left; helical wheel representation of the ALPS peptide [generated with HELIQUEST (Gautier et al., 2008)]. Top right; atomistic starting configuration of ALPS, as adapted from González-Rubio et al. (2011) and overlayed MARTINI beads. Hydrophobic and hydrophilic residues are shown in yellow and magenta, respectively. Bottom; initial (*x* ≈ 27.5 nm) and final (*x* ≈ 5.5 nm) membrane positions on the squeezed 55 × 10 nm^2^ POPC membrane used for umbrella sampling. Scale indicates x-coordinates. **(B)** ALPS free energy profile *F*(*x*) as a function of the x-coordinate. Blue line is the linear fit of the buffer zone, which' slope $\frac{dF\left(x\right)}{dx}$ yields the effective force that acts on the peptide. **(C)** ALPS' membrane thickness-dependent free energy *F*(*a*), calculated for the buffer zone (see Supplementary Material for derivation).

Hereto, we performed umbrella sampling of the ALPS motif of ArfGAP1 along the 10 nm buffer zone of the same 55 × 10 nm^2^ POPC membrane we described before (Figure 3A). This yielded the free energy profile *F*(*x*) with respect to the x-coordinate of the position in the box (Figure 3B, see Figure S3 for histogram). The slope $\frac{dF\left(x\right)}{dx}$ of a linear fit to this energy profile in the buffer zone describes the average attractive force on the peptide toward the thin region of the membrane. For the ALPS motif in the current setup, this force equals 6.89 kJ mol^−1^ nm^−1^, or 11.44 pN. One can interpret this value as a measure for the ability of the peptide to sense lipid packing defects.

Combining the *F*(*x*) profile with the thickness profile *a*(*x*) (Figure 1C, Equation 4) yields the free energy as a function of the membrane thickness *F*(*a*) (Figure 3C, see Supplementary Material for the derivation of the required Jacobian transformation). From this, we could conclude that ALPS indeed favors the thinner section of the membrane due to its ability to sense lipid packing defects. The free energy difference Δ*F*(*a*) between the thin and the normal zone was roughly 25 kJ mol^−1^, or 10 k_BT, which is in the same range as the experimental binding free energy of helical amphipathic peptides to membranes (He and Lazaridis, 2013). This result underlines that the “curvature”-sensing ability of the ALPS motif (and arguably many other proteins) actually boils down to lipid packing defect sensing, and this is consistent with previous simulations (Vanni et al., 2013).

## 4. Discussion

We here presented a protocol for controlled thinning of lipid membranes in MD simulations. Although we performed our simulations with the MARTINI model for CGMD simulations, we should note that our protocol is not at all restricted to coarse-grained systems. We can imagine that, for most applications, the thickness gradient in the buffer zone of the membrane would be the main region of interest. Therefore, one could choose to only simulate that specific area. This would reduce the box dimensions to a system size that is feasible to simulate in atomistic detail (10 × 10 × 10 nm^3^ vs. the current 50 × 10 × 10 nm^3^, for our setup). Since the current work's main goal was to explain the principle of an external thinning potential, we here chose to exaggerate the size of the normal and thin zones for the sake of clarity.

We showed that induced thin zones bear lipid packing defects (Figures 1D,E), comparable to, e.g., membrane regions with a positive curvature. Because of the hydrophobic nature of the thin zone (it is under a net surface tension), it attracts surfactants that are better able to shield the hydrophobic tail region from the water interface. These surfactants generally form membranes with an inherently weaker oil-water surface tension, as evident from their lateral pressure profiles (Figure S1B). As a proof of principle, we illustrated that this way of inducing lipid packing defects can strongly affect the sorting of four different biorelevant lipids (Figures 2B,C). We observed that POPE and cholesterol partitioned *away* from the thin zone of the membrane. With respect to POPC, POPE has a headgroup (N(CH_3)_3 vs. NH_3) that can form inter-molecular hydrogen bonds, thus resulting in stronger homogeneous attractions. This increased homogeneous headgroup attraction is implicitly modeled by the MARTINI model (Marrink et al., 2007) and we indeed observed an increase in POPE headgroup self-interaction upon thinning (Figure S4). Intuitively, POPE sorts away from the thin zone since increased spacing between lipid headgroups is less favorable. It is noteworthy that stronger homogeneous headgroup attractions also increase the surface hydrophobicity of the membrane because this decreases (competitive) heterogeneous interactions with water. As a consequence, PE headgroups have a reduced ability to compensate for the energetic cost associated with the oil-water interface (Figure S1B). This effective increase in surface hydrophobicity simultaneously dictates the molecules' effective shape via the first moment of the pressure profile, which translates into a more negative spontaneous curvature within membrane elasticity models. In line with this shape argument, POPE sorts away from the thin zone where lipid packing defects mimic a positively curved membrane.

Although cholesterol behaved very similarly to POPE in our simulations, additional underlying driving forces are present. Since cholesterol both thickens a POPC membrane and increases its elastic modulus (the membrane becomes “stiffer”) (Daily et al., 2014), diffusing away from the thin zone reduces the equilibrium work required for thinning. In contrast, PE headgroups in fact significantly “soften” lipid membranes within this model, as evident by an increase in bending modulus (Bubnis et al., 2016). Yet, partitioning within the thin zone is unfavorable because PE simultaneously increases surface hydrophobicity.

Conversely, but according to the same principles, LysoPC and PLiPC were *enriched* in the thin zone. For LysoPC, this result is effectively explained by the spontaneous curvature argument (lysolipids decrease surface hydrophobicity because of their small hydrophobic tail volume) and the notion that lysolipids both thin and soften the membrane. The enrichment of the thin zone with PLiPC lipids largely arises from the fact that their highly unsaturated tails are effectively less hydrophobic because alkenes are slightly more hydrophilic than alkanet. As a consequence, PLiPC reduces the hydrophobicity of the membrane surface, as evident by the lateral pressure profile (see Figure S1B). Additionally, PLiPC favors more disordered phases (Pinot et al., 2014) because of its decreased tendency to shield the (less) hydrophobic tails from the water phase. Since membrane thinning reduces lipid order, it is not surprising that PLiPC concentration are elevated in the thin zone.

Qualitatively, our results are consistent with curvature-induced lipid sorting toward regions of a positive membrane curvature (Risselada and Marrink, 2009; Boyd et al., 2017; Baoukina et al., 2018; Elías-Wolff et al., 2018, 2019). For symmetry reasons, sorting in simulations based on membrane curvature are based on a competition between both positive and negative curvature, which can make it hard to distinguish e.g., positive curvature depletion from negative curvature enrichment. Since our system only includes one of both conditions, it can help to deconvolute these effects. For example, PLiPC does not show a pronounced preference for the inner or outer leaflet in small 20 nm-sized lipid vesicles (Risselada and Marrink, 2009), yet we observed a strong sorting toward the thin membrane region. Furthermore, since externally pulled membrane tethers are likely under an additional tension, both curvature and tension driven sorting may occur simultaneously (Baoukina et al., 2018). In such a scenario, differences in the adopted composition between the two monolayers in a tether can alternatively be due to differences in relative stretch between the monolayers.

Beyond lipid sorting effects, we showed that inducing membrane thinning induces sorting of the “curvature” sensing ALPS motif (Figures 3B,C). Umbrella sampling of the ALPS peptide along the buffer zone of the squeezed membrane yielded a free energy difference of ≈ 10 k_BT over a relative decrease in thickness of ≈ 1.1 nm. These obtained values are similar to the binding free energies that were experimentally determined for such peptides (He and Lazaridis, 2013). The slope of the free energy profile along the buffer zone equates to the attractive (or repulsive) force on the peptide toward the thin zone of the membrane. For ALPS, this attractive force was estimated to be about 11 pN–a substantial biomolecular force close to, for example, the force exerted by a neuronal SNARE complex (Gao et al., 2012). Such a force allows for direct, quantitative measuring of lipid packing sensing ability and could be exploited to validate, score, and rank the sensing ability of amphipathic molecules. Since the obtained free energy gradient, $\frac{dF\left(x\right)}{dx}$, is rather linear, it already suffices to restrict sampling to only a handful of points along the buffer zone, or obtain $\frac{dF\left(x\right)}{dx}$ even from a single simulation. Hence, $\frac{dF\left(x\right)}{dx}$ is simply the average force acting against the umbrella potential. This means that our protocol can serve as a very efficient way to both quantify and score protein sorting effects.

Taken together, we have shown that our thinning protocol is able to induce lipid and protein sorting in line with theory and previous simulations and experiments. Since the driving force (sensing lipid packing defects) for such thinning-induced sorting is the same as in, for example, membrane curvature and membrane junctions, our protocol can serve as a proxy for studying such phenomena *in silico*. Moreover, and for the first time, it allowed us to “measure” the attractive force acting on the ALPS sensor motif toward lipid packing defects. Finally, because of the protocol's efficient and tunable nature, it enables high-throughput scoring of lipid packing-induced sorting effects of virtually any amphipathic molecule.

## Data Availability Statement

The code used in this study can be found at https://github.com/nvanhilten/thinning_protocol.

## Author Contributions

NH and HR designed the experiments and implemented the thinning protocol code. NH performed all MD simulations and analyzed the data. KS contributed to the free energy calculations. NH and HR wrote the manuscript.

### Conflict of Interest

The authors declare that the research was conducted in the absence of any commercial or financial relationships that could be construed as a potential conflict of interest.
