# Supplementary material for: Membrane Thinning Induces Sorting of Lipids and the Amphipathic Lipid Packing Sensor (ALPS) Protein Motif
Source: Front Physiol. 2020 Apr 16;11:250. doi: 10.3389/fphys.2020.00250 (PMC7177014; doi:10.3389/fphys.2020.00250)
Supplement: Supplementary file 1 [file Data_Sheet_1.PDF]

## Supplementary Material

### LATERAL PRESSURE PROFILES

Compared to a tensionless membrane (black line in Fig. S1A), the pressure profile for a membrane under tension (e.g. through external thinning, red line in Fig. S1A) shifts down; an increase of lipid packing defects. When curving a membrane (e.g. vesicle, schematic blue dashed line in Fig. S1A), the oil-water surface tension peak gets less strong for the inner leaflet (negative curvature) and proportionally stronger for the outer leaflet (positive curvature) (Ollila et al. (2009); Nepal et al.(2018)), along with an increase of lipid packing defects. This reasoning illustrates the aim of our thinning protocol: we mimic positive curvature (drop of oil-water surface tension peak in pressure profile) by locally squeezing the membrane.

From the lateral pressure profiles of pure POPC, PLiPC and POPE membranes (Fig. S1B), it is evident that POPE gives a stronger (more negative) and PLiPC gives a shallower oil-water surface tension peak.

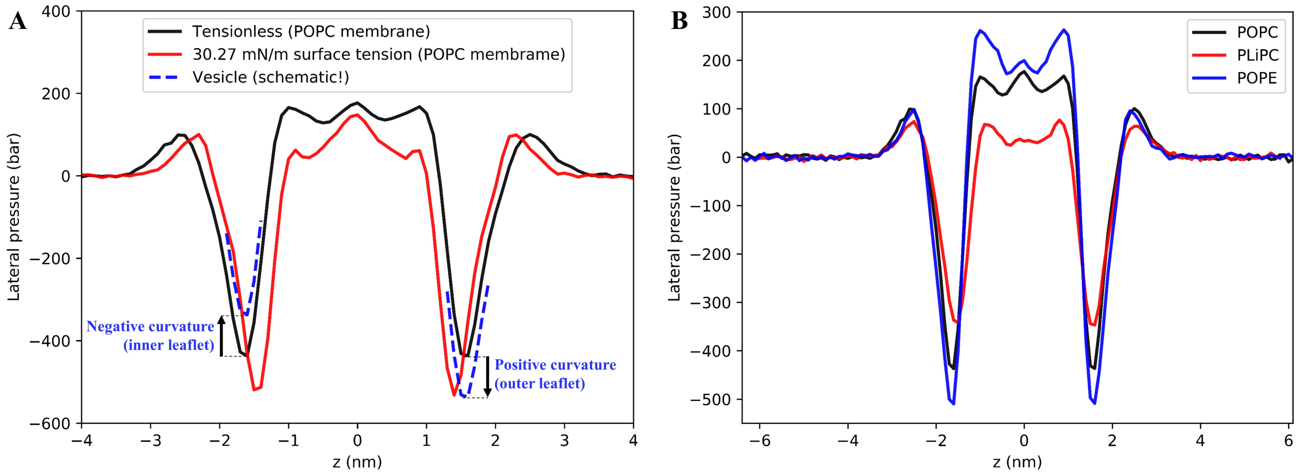


**Figure S1:** **(A)** Lateral pressure profiles of a tensionless POPC membrane (black line), a POPC membrane under tension (red line) and a schematic representation of a vesicle pressure profile (blue dashed line). **(B)** Lateral pressure profiles of a pure POPC membrane (black line), a pure PLiPC membrane (red line) and a pure POPE membrane (blue line).

## SURFACE TENSION CALIBRATION

Applying the thinning potential to the center area of the bilayer results in a net non-zero surface tension. For the system to be stable, one needs to compensate this by coupling the system's pressure to an external lateral surface tension (ST), chosen such that the 'non-squeezed' normal zone is effectively tensionless and, as a consequence, adopts the same thickness as a well equilibrated membrane (semiisotropic pressure coupling to 1 bar, with the same lipid composition) would adopt in the absence of the thinning potential. We did this calibration by running short (20 ns) simulations at lateral STs ranging from 25 to 50 mN/m and measuring the resulting thickness of the normal zone. Linear fits yield the appropriate surface tension values for a given equilibrated membrane thickness (Fig. S2). When applying the resulting surface tensions in thinning equilibration runs, the thickness of the non-squeezed normal zones indeed closely matches the thickness of a NPT-equilibrated membrane without external thinning (Table S1).

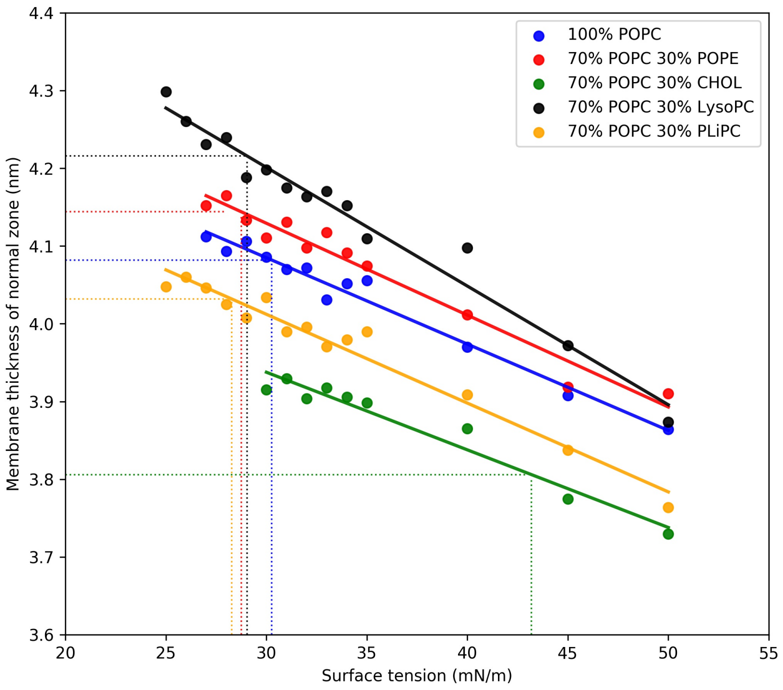


**Figure S2:** Calibration of the applied surface tension (ST) coupling and the resulting thickness of the normal zone of the 55x10 nm$^2$ POPC membrane. Linear equations were fitted to the data. 100% POPC; $-0.0111 \cdot ST + 4.41829$ ($R^2 = 0.974$). 70% POPC 30% POPE; $-0.0182 \cdot ST + 4.48383$ ($R^2 = 0.962$). 70% POPC 30% CHOL; $-0.0100 \cdot ST + 4.23726$ ($R^2 = 0.951$). 70% POPC 30% LysoPC; $-0.0153 \cdot ST + 4.65925$ ($R^2 = 0.968$). 70% POPC 30% PLiPC; $-0.0114 \cdot ST + 4.35497$ ($R^2 = 0.963$). By plugging in the thickness of a well equilibrated membrane (horizontal dotted lines), the calibrated surface tension coupling is found for each lipid composition (vertical dotted lines, see bold values in Table S1).

**Table S1:** Membrane thickness of the normal and the thin zones and box length ($average \pm STD$ over five runs) for different membrane compositions. Semi = semiisotropic, ST = surface tension. Bold surface tension values correspond with x-axis intercepts of the dotted lines in Fig. S2.

| Composition | Mode | P-coupling | Thickness normal (nm) | Thickness thin (nm) | Box length (X, nm) |
|---|---|---|---|---|---|
| 100% POPC | No Thinning | Semi; 1 bar | $4.082 \pm 0.049$ | - | $51.58 \pm 0.02$ |
| 100% POPC | Thinning | ST; **30.27 mN/m** | $4.080 \pm 0.014$ | $2.926 \pm 0.011$ | $55.36 \pm 0.03$ |
| 70% POPC 30% POPE | No Thinning | Semi; 1 bar | $4.144 \pm 0.077$ | - | $50.99 \pm 0.02$ |
| 70% POPC 30% POPE | Thinning | ST; **28.76 mN/m** | $4.148 \pm 0.023$ | $2.947 \pm 0.011$ | $54.54 \pm 0.04$ |
| 70% POPC 30% CHOL | No Thinning | Semi; 1 bar | $3.806 \pm 0.108$ | - | $45.56 \pm 0.03$ |
| 70% POPC 30% CHOL | Thinning | ST; **43.18 mN/m** | $3.816 \pm 0.034$ | $2.586 \pm 0.035$ | $50.25 \pm 0.08$ |
| 70% POPC 30% LysoPC | No Thinning | Semi; 1 bar | $4.216 \pm 0.018$ | - | $48.08 \pm 0.02$ |
| 70% POPC 30% LysoPC | Thinning | ST; **29.03 mN/m** | $4.213 \pm 0.014$ | $2.961 \pm 0.014$ | $51.66 \pm 0.05$ |
| 70% POPC 30% PLiPC | No Thinning | Semi; 1 bar | $4.032 \pm 0.018$ | - | $52.00 \pm 0.03$ |
| 70% POPC 30 % PLiPC | Thinning | ST; **28.27 mN/m** | $4.047 \pm 0.011$ | $2.930 \pm 0.012$ | $55.62 \pm 0.03$ |

## LIPID MIXING MOVIES

We provided movies of 4 μs simulation trajectories (one frame every 10 ns) that show lipid demixing in squeezed membranes of different lipid composition (pure POPC, 30% POPE, 30% CHOL, 30% LysoPC and 30% PLiPC). In all movies, 70 mol% POPC is shown in transparent gray and 30 mol% of mixed-in lipid is shown in orange.

# UMBRELLA SAMPLING HISTOGRAM

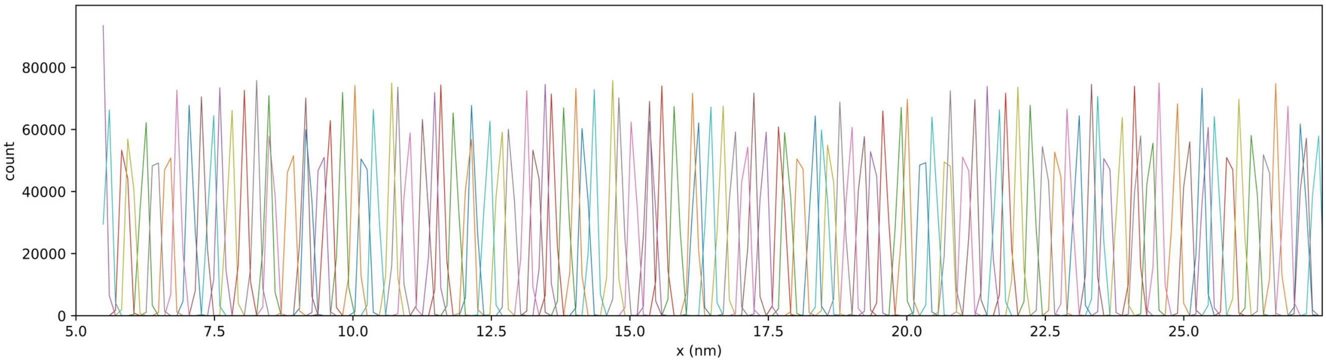


**Figure S3:** Histogram for the 111 windows used in umbrella sampling of the ALPS peptide along the buffer zone of the squeezed 55x10 nm$^2$ POPC membrane.

## DERIVATION OF THICKNESS-DEPENDENT FREE ENERGY

Umbrella sampling was used to calculate the free energy profile for the ALPS peptide motif as a function of the x-coordinate $F(x)$, that is by definition given by:

$$F(x) = -k_{\mathrm{B}}T \ln P(x) \tag{S1}$$

Since we are interested in the free energy as a function of membrane thickness $a$, we need to take into account that the probability distribution $P(a(x))$ changes when we change the variable:

$$P(a(x)) = P(x) \left| \frac{dx}{da} \right| \tag{S2}$$

For the free energy, this Jacobian transformation leads to:

$$\begin{aligned} F(a(x)) &= -k_{\mathrm{B}}T \ln P(a(x)) = -k_{\mathrm{B}}T \ln \left( P(x) \left| \frac{dx}{da} \right| \right) \\ &= -k_{\mathrm{B}}T \ln P(x) + k_{\mathrm{B}}T \ln \left| \frac{da}{dx} \right| = F(x) + k_{\mathrm{B}}T \ln \left| \frac{da}{dx} \right| \end{aligned} \tag{S3}$$

Plugging in the sigmoid function $a(x)$ we used to fit the thickness profile of the buffer zone (eq. 4 in main text) yields:

$$F(a(x)) = F(x) + k_{\mathrm{B}}T \ln \left| \frac{da}{dx} \right| = F(x) + k_{\mathrm{B}}T \ln \left| \frac{L\kappa e^{-\kappa(x-x_0)}}{\left(1 + e^{-\kappa(x-x_0)}\right)^2} \right| \tag{S4}$$

## POPE HEADGROUP RDF

We plotted the 2D radial distribution function (RDF) for POPE and POPC headgroup amine beads ($NH_3$ versus $N(CH_3)_3$) for 2µs trajectories of equilibrated 55x10 $nm^2$ membranes with and without thinning (averaged over the two leaflets). Here, one can see that POPE headgroup self-interactions (black lines) are dominant over heterogeneous interactions with POPC (red lines) upon thinning.

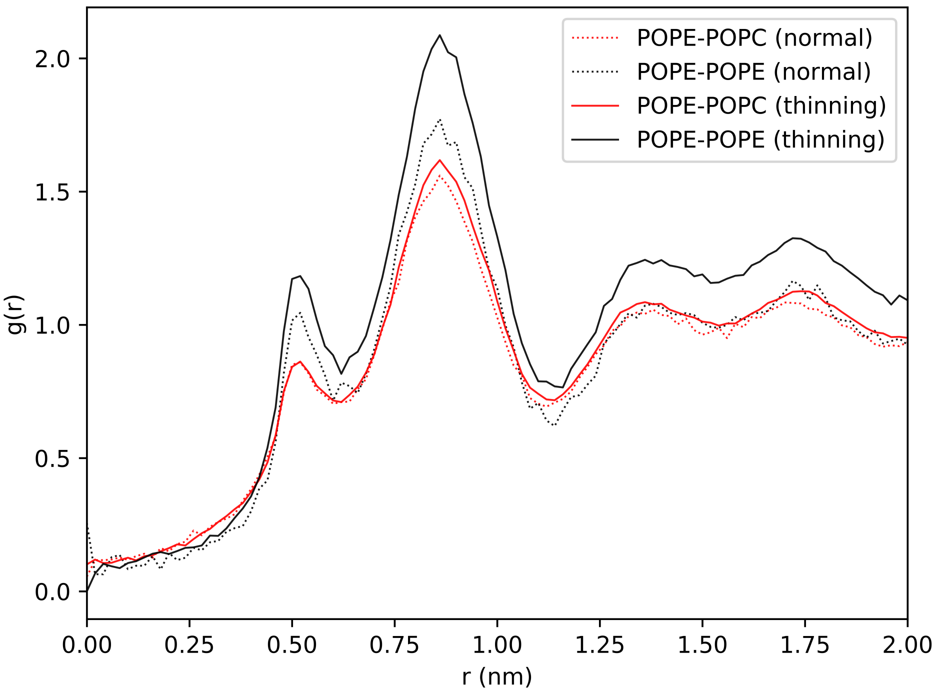


**Figure S4:** 2D RDF plot for POPE and POPC headgroup amines in 2µs of equilibrated 55x10 $nm^2$ membranes with (solid line) and without thinning (dotted line).
